# Divergent Views and Experiences Regarding ‘Completed Life’ and Euthanasia in the Netherlands

**DOI:** 10.1177/00302228211066681

**Published:** 2021-12-28

**Authors:** Priya Satalkar, Sjaak van der Geest

**Affiliations:** 1 Bioethics Institute Ghent, Ghent University, Ghent, Belgium; 2 Department of Medical Anthropology, 100440 University of Amsterdam, Amsterdam, The Netherlands

**Keywords:** ageing, completed life, euthanasia, tiredness of life, views and experiences, the Netherlands

## Abstract

A small proportion of older people in the Netherlands want to end their lives because they feel their lives to be ‘completed’ and no longer worth living. Currently, there is heated debate over whether or not these people should have the right to euthanasia. Drawing on previous research, we conduct a heuristic analysis of views and experiences of three different ‘parties’ involved in this debate, namely, the older people, their relatives and friends and medical professionals. The views of these three groups tend to be divergent and conflicting, posing a difficult dilemma to decision-makers.

## Introduction

For about half a century there has been wide-spread support for euthanasia under certain conditions in The Netherlands (Kennedy, 2002). In the Dutch context, *euthanasia* is defined as the deliberate ending of a person’s life, at the person’s explicit request, by a physician. *Physician-assisted suicide* (PAS) is defined as the person self-administered medication that is prescribed by a physician. For reasons of readability, in this article, we will use the term ‘euthanasia’ to mean both.

In 2002, the Dutch government issued the ‘Termination of Life on Request and Assisted Suicide (Review Procedures) Act’, which states that euthanasia will be permitted under certain conditions: there must be unbearable suffering without prospect of improvement and an explicit, well-considered request from the patient. A second independent physician must be consulted as well (Dutch Ministry of Security and Justice, 2002). Patients, however, do not have a *right* to euthanasia. Physicians are not under any obligation to perform euthanasia. They have a right to conscientious objection (De Jong & Van Dijk, 2017).

Most requests for euthanasia come from people whose suffering is unbearable and who regard a self-chosen death to be the only way out. The suffering almost always has a physical basis. To illustrate: in 2018 the Regional Testing Committees for Euthanasia received 6126 notifications of euthanasia. This is 4% of the total number of people who died in the Netherlands in that year (153,328). In 65.5% of cases (4013 in total), the requests concerned cancer patients in the final stages of their illness (Regional Euthanasia Review Committee, 2018).

After many years of practice, there is wide acceptance among the Dutch public for the current euthanasia law (Schnabel et al., 2016). Among physicians, there is also broad consent for the current practice, especially in cases with an evident medical basis. Research demonstrates that a large majority of Dutch physicians find it conceivable that they would grant a request for euthanasia made by a patient with cancer (85%) or another physical disease (82%) (Bolt et al., 2015). In more complex cases, however, the potential for consent decreases: only about one-third of physicians (34%) would find it conceivable that they would grant a request for euthanasia by patients with psychiatric illnesses, and about one-fourth (27%) in cases of tiredness of life (Bolt et al., 2015). Although in practice the vast majority of patients who receive euthanasia is still terminally ill, euthanasia has also been legally performed on an increasing number of patients with dementia, psychiatric disorders or multiple geriatric syndromes (Evenblij, Pasman, Van Der Heide, Hoekstra, & Onwuteaka-Philipsen, 2019). It follows that the public debate has changed and now focuses on these complex cases as well as the interpretation of the legal criteria for euthanasia in such cases. Three main topical issues spark considerable debate:

1. People who have advanced dementia are often excluded from the right to euthanasia, even if they have an Advanced Euthanasia Directive (AED). Although an AED can legally replace direct communication, in practice most physicians experience problems during the decision-making process. These problems mainly concern the evaluation of the due care criteria, that is, whether the wish is voluntary and well-considered and whether the patient’s suffering is unbearable (Schuurmans et al., 2019).
2. At present, one of the due care requirements is that the physician has to be convinced that the patient’s suffering is both unbearable and hopeless, without any future perspective. With regard to the latter point, in dialogue with the patient, the physician has to take into account the treatment options available. The question of whether the suffering is unbearable should in first instance be answered by the patient, based on his own experience (Dees et al., 2011). Although the patient’s opinion is very relevant, the physician involved also has to be convinced that the suffering is unbearable. Consequently, the patient has no authoritative voice and is dependent on the physician’s final judgement.
3. Some people may want their life to end because they come to the conclusion that the quality and the meaning of their life has deteriorated to such an extent that they prefer death over life while the suffering has no medical basis. Based on current jurisprudence, however, unbearable suffering must have a medical dimension in order for euthanasia to be legal (Hoge Raad, 2002; Regional Euthanasia Review Committee, 2018, 53).

The final two points are relevant for the topic of this study, in particular the request for euthanasia based on the consideration of having lived a ‘completed life’ (in Dutch: ‘*voltooid leven’*). In this article, *our purpose is to conduct a heuristic analysis of statements about completed life euthanasia to better understand divergent views.* We thus hope to contribute to a future research agenda which addresses the different interests and concerns at stake. To put this in context, we first sketch the historical and social background and provide some information about our empirical work.

## Background

In 1991, Prof. Huib Drion, a Dutch Supreme Court judge and professor of civil law, laid out an argument in a newspaper article that many older people are looking for an acceptable way to step out of life when they feel tired of it and no longer want to live (Drion, 1992). He made a plea for a means to realize a self-chosen death that people could have at their disposal and could take without involving a doctor; this became known as the so-called Drion pill. Drion’s proposal did not directly lead to policy decisions, but it did draw public attention to self-euthanasia and assisted self-chosen death as a possibility, even in the case of tiredness of a life that was felt to be completed.

In 2002, the Dutch Supreme Court ruled that euthanasia may not be carried out if the suffering of the patient is not mainly determined by a medically classifiable disease. This verdict was reached in the context of the Brongersma case, which involved a physician-assisted death carried out for an 86-year-old man who was tired of life. In 2004, the Royal Dutch Medical Association (KNMG) published a report on the role of physicians in dealing with people ‘suffering from life’ (Commissie Dijkhuis, 2004). This commission proposed a broader medical domain than had been determined by the Supreme Court. They expressed the opinion that the legal demarcation of a medical cause does not always reflect the complexity of medical practice and that tiredness of life should not automatically be placed outside of the medical domain (Commissie Dijkhuis, 2004).

In 2010, the discussion about ‘completed life’ became even more prominent with the launch of a petition by a group calling themselves *Uit Vrije Wil* (Of Free Will), which was signed by 116,871 citizens (Peters et al., 2011). The petition advocated for an amendment to the existing legal framework for euthanasia to create the possibility of a dignified death for Dutch older citizens, based on the principle of self-determination and free will. In the petition, the concept of ‘completed life’ applied to citizens 70 years or older who have a consistent and well-considered wish to die, in the absence of unbearable suffering due to medical illness. At that time, Dutch law did not acknowledge such requests, although several authors had argued that the existing law could be stretched in its interpretation to include such requests (Ost & Mullock, 2011).

The petition’s aim was to arouse a public debate about the existing euthanasia law. It was discussed in the House of Representatives after a motion calling on the government to involve the citizens’ petition in its evaluation of the euthanasia law. The government’s response was to state that the citizens’ petition did not relate well to the current system, but that further consideration of the issues it raised was important. The government thus decided to conduct a study of the legal possibilities and social and ethical dilemmas of euthanasia for people who deem their life to be complete. An advisory committee was installed to carry out this study.

In January 2016, the advisory committee returned with its advice, recommending against any changes of or additions to the current law. The committee also remarked that the number of people who wish to die while in a relatively good state of health is probably very small (Schnabel et al., 2016). Furthermore, they argued that the wish for euthanasia usually comes with an accumulation of physical and/or mental complaints in old age and they therefore recommended further exploration of the existing possibilities within the current law to perform euthanasia in such cases. They also highlighted the risks of additional legislation, namely, the possibility of undermining the current euthanasia practice, creating social pressure on older people, as well as the possibility of reinforcing the stigma of old age (Schnabel et al., 2016).

Nine months later, however, in October 2016, two government ministers, apparently ignoring the advice of the advisory committee, proposed that new legislation should be made to help older people who wish to die after a ‘completed life’ (Schippers & Van der Steur, 2016). In reaction to this proposal, the Dutch liberal party ‘Democrats 66’ (D66) took it one step further and launched a detailed draft bill in line with the ministers’ proposal. The guiding principle in this new law would be autonomy rather than compassion. The focus on the role of the physician and his/her conflict of duties (preserving life vs. alleviating intolerable and hopeless suffering) was accordingly shifted to a focus on the right of citizens to self-determination and the right to die when they wish (D66, 2016). These proposals once again sparked passionate debate both for and against and proved to be a thorny issue in the formation of a new government following the 2017 election. The new government included a Calvinist political party (SGP) that was opposed to any form of euthanasia and completed life euthanasia in particular. The government, therefore, postponed a political decision on the issue and changed the focus by calling for more public debate and commissioning further research. The discussion is continuing at the time of writing this article (November 2021), while political parties attempt to form a new government.

In January 2020 the results of further research were presented to the Minister of Health, Welfare and Sport. More than 21,000 elderly people (above the age of 55) had completed an extensive questionnaire while the researchers had also interviewed dozens of elderly people in-depth. More than 200 executed and rejected euthanasia requests had been analysed. Finally, 1600 GPs also participated in a short questionnaire survey (Van Wijngaarden et al., 2020).

The research estimated that 0.18% of all people aged 55 and over have a wish to end their lives without being seriously ill. That amounts to just over 10,000 people in the entire country. More than a third of this group would like to get help with suicide. Two thirds preferred to end life by themselves. Just as before the Advisory Committee Schnabel, the report emphasized that the term ‘completed life’ was inappropriate, because it suggested a too rosy picture of the state in which these older people requested euthanasia and obscured the reality leading to that request. That reality included, for example, emotional suffering, loneliness, poverty and the fear of total dependency and undignified death. The overall policy suggestion that public media took from the report was that a new law to facilitate ‘completed life euthanasia’ would not be a good idea. Meanwhile, the D66 political party announced that it would soon present a proposal for such a law.

Although the legal and ethical aspects of the Of Free Will petition (2010), as well as its impact on policy and legislation, have been frequently addressed, both in the media and in the academic literature, much remains unclear about the ideas of the various actors in this discussion. ‘Completed life’ as a reason for voluntary death is a murky issue, full of contradictions and divergent claims. In this article, we focus on the views of three main ‘parties’ that tend to have different ideas about this type of voluntary death: (1) older people (in this article, divided into those with an actual and urgent death wish and those who endorsed the Of Free Will petition and expressed a prospective wish for ‘completed life euthanasia’); (2) their relatives and friends and (3) medical professionals. We use these three groups as heuristic categories, with the aim of clarifying the tensions between their divergent views and suggesting a future research agenda which addresses the different interests and policy concerns at stake. Obviously, the described groups and their views should not be understood as fixed and mutually exclusive entities. Indeed, we are aware that there is not only tension between the different stakeholders but also within each of them and even within individuals (Van Wijngaarden et al., 2015; 2016b).

## Methods and Data

For this study, we performed a ‘heuristic analysis’ which is an anthropological qualitative way of exploring the complexity of a situation, event or institution by studying a relatively small number of participants. This small scale allows for an in-depth analysis that is likely to detect little known and unknown processes. The ‘discoveries’ can then be integrated in a larger and possibly more quantitative research. ‘Heuristic analysis’ can thus be seen as an attempt to find the right questions before setting up an elaborate research project. For our heuristic analysis, we draw on previous phenomenological research conducted by Els van Wijngaarden and colleagues among older people who expressed the belief that they had reached the stage of ‘completed life’ (Van Wijngaarden et al., 2015; 2016b). This is supplemented with previous research conducted by the first author among supporters of the ‘Of Free Will’ petition for ‘completed life euthanasia’ (Satalkar, & Van der Geest, 2019).

Van Wijngaarden et al. (2015, 2016b) carried out a qualitative research project using a phenomenological or ‘lifeworld’ perspective (Dahlberg et al., 2008). Their aim was to capture the views and lived experiences of older people confronted with the acute feeling that their life was complete. The participants were 25 Dutch mentally competent older people (67–99 years old, mean age 82 years) without evidence of a serious physical or mental illness. The 25 participants were recruited through research advertisements in various magazines targeting older people who wished to die because they felt their life was completed.

Open interviews were conducted to study the phenomenon from an insider perspective. Emphasis was not placed on perceptions and thoughts, but on the way in which the phenomenon was lived in everyday life. The interviews took place from April to December 2013 in participants’ own homes and lasted 2 hrs on average. The interviews were audio recorded and transcribed verbatim. The various publications that have resulted from this research are based on diverse research analysis techniques such as reflective lifeworld analysis, thematic analysis, metaphor analysis and a case study of one older couple (Van Wijngaarden et al., 2015a, 2016a, 2016b, 2017).

The exploratory study by the first author of the current article was conducted in 2011, shortly after the launch of the ‘Of Free Will’ petition. She aimed to understand the reasoning and decision-making of individuals who had endorsed the petition. The semi-structured in-depth interviews (or conversations) were carried out among nine Dutch citizens living in the western part of The Netherlands, six women and three men (between 48 and 70 years). Seven of the nine respondents had a university degree. During the conversations, ideas around free will and decision-making were central to the respondents’ reasoning. Each conversation lasted between 90 and 120 min. All were audio recorded and transcribed verbatim. The transcriptions were sent to the respondents for their feedback, clarification and comments. Data analysis was done by the researcher and the second author independently. Consensus was sought in cases where they reached differing interpretations. The second author also provided comments throughout the research and writing period.

The choice of these two research projects was based on the fact that both Van Wijngaarden et al. and Satalkar provided in-depth qualitative insights in people’s views concerning completed life euthanasia, but among two different categories of respondents. This made it possible to investigate and compare views and claims of older people who were faced with the immediate wish for assisted dying in case of ‘completed life’ and those for whom the wish for an assisted death was a more distant future possibility; in short; we studied urgency versus political standpoint.

Both Satalkar and Van Wijngaarden interviewed only people who were reflecting on their wish to die (either current or prospective) based on the experience of having lived a ‘completed life’. As a result, the ideas of relatives and medical professionals were less discussed in the ensuing publications. In this article, for the perspectives of relatives, we use quotes in which the older people reflected on the roles and concerns of close family members and friends. Reading and interpreting these quotes, we should always keep in mind that they originated from the perspective of the older people themselves and should thus be considered ‘second-hand’. To supplement our data on the perspectives of medical professionals, we reviewed additional publications and reports dealing with medical views and concerns on ‘completed life euthanasia’ (Bolt et al., 2015; Schnabel et al., 2016; Schothorst, 2015; Van Delden et al., 2011).

## Results

The conflicting views that we discuss in this paper are first of all conflicts between different sorts of experience depending on different positions in relation to voluntary death wishes. In what now follows, we discuss the divergent views of three groups of actors involved in discussions on ‘completed life euthanasia’, namely, older people, their relatives and friends and medical professionals.

### The Views of Older People

As mentioned above, by ‘older people’ we mean here both the interviewees of Van Wijngaarden and colleagues who were in fact older persons and felt that their life was complete, as well as the interviewees of Satalkar who had signed the ‘Of Free Will’ petition, who were of a generally younger age, but who in the interviews talked about themselves in a possible future, when they imagined being in the position of wishing to die because of a ‘completed life’. The former interviewees (by Van Wijngaarden), who had a death wish due to the consideration that their lives were complete and no longer worth living, expressed their feelings in vivid terms and seemed determined to make their wishes come true. Here are two quotes:

My children are all managing on their own. Nobody lives a shabby life, thank God. But you know, they don’t need me anymore. I know I’m not supposed to say it out loud, but when I visit my kids, I think they would say: ‘God damn, it’s the old man again’ … I’m just getting in their way [man, aged 83].^1^ (Van Wijngaarden et al., 2015, p. 260).

You have no effect on anything, you know. The ship sets sail and everyone has a job, but you just sail along. … I am a burden to them. That’s not easy. Not easy. No, no, no. That’s not me! No, no, no, no, no! … Yeah, it is difficult to fully express, huh, what I’m feeling. Humiliation is too strong a word, but it is bordering on it. Huh, I simply feel ignored, completely marginalized [man, aged 92]. (Van Wijngaarden et al., 2015, p. 261)

The majority did not elaborate much on how they would realize their death wish, but most seemed to hope for a legal option.

I just want to have the space and the freedom [for a legal assisted death]. I have organized everything and accurately described my wishes for euthanasia. Really, there is no need for somebody to be punished. I just want to get assistance in a dignified way. So that I don’t need to hang myself or jump from a bridge (woman, aged 84).

I think that the law should change, because you know, formerly, it used to be much more usual, at least in some cultures. With the Eskimos, for example: if older people felt that they could no longer keep up with the rest, they said: ‘My strength has deteriorated and I don’t want to be a burden’. And then they walked into the perpetual snowfields. My whole life, I’ve been thinking about this without any dramatic feeling. It’s so logical to me [woman, aged 74].

While these older people who were tired of life were quite determined about their wish for a self-chosen death, Van Wijngaarden and colleagues nevertheless observed conflicting ideas about voluntary death, as well as about the complexity of decision-making regarding such a death. On the one hand, the interviewees claimed that they were making an autonomous decision and were personally in control of everything, but on the other hand they admitted to being uncertain about their own considerations, about the assistance of doctors, as well as about the support of their loved ones. Van Wijngaarden and colleagues’ study of the period *in between* intending and performing a self-directed death reveals a series of profound paradoxes:

Participants’ accounts are permeated with ambivalences and ambiguities. They felt both detached and attached; they felt both ready to give up on life and yet tending to postpone hastening death; they sensed both their wish to die was sound and rational and simultaneously they felt driven by much more uncontrolled compulsions and they took all efforts to organize a ‘good death’ but nevertheless were permeated by uncertainties and worries as they realized their impossibility to fully control death (Van Wijngaarden et al., 2016b, p. 8).

Van Wijngaarden et al. (2015: 260–262) describe five aspects of the ‘completed life’ experience: (1) a sense of aching loneliness; (2) the pain of not mattering; (3) the inability to express oneself; (4) multidimensional tiredness and (5) a sense of aversion towards dependence.

A sixth aspect, closely related to the fifth above, is the anticipated fear of a miserable, painful and dehumanizing end of life, as we will see in the statements of Satalkar’s research participants. These interviewees wanted to prevent unbearable suffering due to physical and mental decline by being able to opt for a self-chosen death at a time right for them.

I have an aunt who is 97. She is in a care home. A nurse has to help her out of bed, put her in bed. The whole day she sits in her chair. She sometimes can’t remember me. She’s completely stooped [shows me how stooped this aunt is]. I don’t want that [firm tone of voice]… When I see my aunt in her current situation, I’m so happy that I won’t suffer like her because I’m not bound by strong catholic belief like she is. You can have a strong wish to die even when your heart is beating and kidneys are functioning. I don’t want to die like a baby, as I was born. Walking with support, incontinent, stooped, unable to talk, unable to feed myself, saliva drooling from my mouth. I don’t want to die like that. [woman, aged 65].

Life is about hope and expectation. Life is about projecting yourself into the future. There can be all sorts of reasons where there is no projection of yourself and your life in the future. Maybe because you’re just too old to be still interested in life. We had this documentary on TV with a 98-year-old woman who said, ‘I love my children and grandchildren. My granddaughter is pregnant with a baby. I’m simply not interested anymore, although I love them’. It takes life energy to project yourself as a grandmother with another new baby. If that has faded away, then you can imagine that someone says, ‘This is enough!’ [woman, aged 64].

It is significant that the nine interviewed signatories of the ‘Of Free Will’ petition were more radical and outspoken in their visualization of their future voluntary death than the interviewees in the study of Van Wijngaarden and colleagues. They were less concerned about professional medical assistance, while some were even squarely against professional interference, as the following quote shows.

I don’t want to be dependent on the medical profession. I don’t agree with the fact that the doctors decide whether I should live for another week because they don’t want to pull the plug [life support]. We live longer, not just because of us but because the doctors keep us alive. That’s not my decision, it’s *their* decision… My argument for the Initiative [petition] is that I have had this independent life. I don’t want to be dependent on the doctors to decide when my time is coming [woman, aged 64].

The interpretation of what constitutes a ‘completed life’ did not differ much between the older people interviewed by Van Wijngaarden and colleagues and those who had signed the ‘Of Free Will’ petition in advance of their old age anticipating the possibility of finding themselves in such a condition. Ideas about the *way* in which this ‘completed life’ should be completed (ended), however, differed significantly between the two groups. The most likely explanation for this difference is, in our view, the fact that the signatories of the petition were not yet facing the immediate need to decide. They could afford a more radical opinion, since the moment of acting was still far off. Their standpoint had a more comfortable ‘armchair’ character, one could say. Their responses were focused on advocating for the legalization of self-directed death, based on politically driven, ideological opinion, combined with observations other people’s lives. The accounts of the older people with an actual and current wish to die, however, were mainly based on firsthand experiences. They were standing at the threshold of taking the final step and were more cautious and ambivalent. They were struggling with a constant sense of being torn, expressed in words such as ‘dilemma’, ‘quandary’, ‘split position’, ‘unsolvable problem’ and ‘living in two minds’. The ‘knowledge’ that both groups claimed with regard to their position and agency clearly differed according to their situation: urgency and actuality versus anticipation and still having time.

### The Views of Relatives and Friends

Except for two cases (described below), the children, other relatives and friends of older people who wished to end their lives because they considered them to be ‘complete’ were not directly interviewed. Their reactions to their loved ones’ desire for ‘completed life euthanasia’ reached the researchers mainly through the conversations with the older people themselves, when Van Wijngaarden and colleagues asked them about their decision-making processes and death wish.

One participant (man, aged 93), quoted above, said that his wife had a ‘*totally different opinion’* about the end of life than he had, so he had to wait. Another participant (woman, aged 87) told the interviewer that she had invited a volunteer helper for lunch in a cozy restaurant. She then said to her: ‘*Perhaps this is the last time together’*. The helper became a bit angry and replied: ‘*You must not say this!’* Another participant (man, aged 83) was deeply concerned about the reactions of his children: ‘…*If they all show the same emotions as my daughter, I don’t think I can handle it. Then I’ll probably give up my freedom to decide on my own life*’.

The only instance in which children were officially interviewed was in the case of a couple who were planning to die together because they felt tired of life. The children of this couple found themselves in a big dilemma. On the one hand, they felt they needed to respect their parents’ wishes, but on the other hand, they struggled intensely with their plans. Their son complained:

I feel powerless, because we never really talk about it. … OK, we did talk about it, actually quite often. … But always very rationally and very abstractly. … But we never talked about the emotional side of things. … They apparently believe that their life is over. Though we, as children, totally disagree with this idea. (Van Wijngaarden et al., 2016a: 1069).

The son of another participant (woman, aged 74) held a somewhat different view, saying that for him a self-chosen death was ‘*absolutely no taboo’*. He emphatically positioned himself as being free-thinking and a great supporter of freedom of choice around the end of life. But when it came to a more personal, rather than abstract, level, he revealed more ambivalence: ‘*My mother? Already? That’s just way too early!’* (Van Wijngaarden, 2016a).

None of Van Wijngaarden and colleagues’ 25 interviewees spoke about children and other people around them who supported them unconditionally in their death wish. While most older people wanted to end their lives in good harmony, openness and dialogue with their loved ones, in many cases they experienced that their loved ones had intense difficulties with their death wish. Often, opinions about the right to die at a self-chosen moment had been exchanged and were accepted, but when things became real and concrete, it often turned out to be incredibly difficult to talk about the underlying emotions. Children found it difficult to listen to their parents, and parents could not give room to the emotions of their children. Indeed, such discussions brought up a lot of friction in the relationship. Often, this had to do with the fact that family members were shocked because they viewed their parents’ death wish as untimely.

It would be premature, however, to conclude from this that the older people received no support from their friends and relatives and only faced criticism and disapproval from them. As stated before, we should consider the fact that the role of relatives and friends was not the focus of the interviews, so it is not surprising that the older people only spoke about matters that were troubling them. What can be concluded with certainty, however, is that the attitudes and reactions of relatives and friends are likely to have a strong influence on the experiences and decisions of older people when it comes to seeking an assisted death after having lived a ‘completed life’. This conclusion needs further research.

Again, a comparison with Satalkar’s nine respondents who signed the ‘Of Free Will’ petition may be instructive. Seven of them had some sort of conversation with relatives or friends before signing the initiative. They met support as well as disagreement, but the matter never became highly emotional since the decision had no direct consequences for anyone. The signatories emphasized however that after having completed all of their responsibilities towards their family, they had the right to decide on the moment and way of dying by themselves. The following conversation excerpt between S (Satalkar) and W (woman, 65 years old) illustrates this.

S: What is dignified death?

W: Dignified is not jumping out of a window or in front of a train. Dignified death is when you are surrounded by your loved ones and you take your last drink and your family is comfortable with your decision.

S: Would you have changed your mind about [supporting] the initiative if your daughter had a strong opposition to these views? Would it have changed your decision?

W: I think that it would have made some difference. But I would have said to her that it is my life and I have to decide, even if you do not agree with me. I understand your wishes and sorrow. But I suffer so much and at some point, you have to live without me anyway.

S: … But if you have to go against your family to decide what you want, is it not a lonely position?

W: I cannot believe that if the children see how their mom is suffering that they won’t agree. If they do that, then it is very selfish on the part of the children to keep their mom alive who has nothing to live for and who is suffering. It is pure selfishness of the children. But that is my idea. When I signed the initiative, I was very clear that it is what I wanted. In fact, it was something that I was really looking for.

Of course, as mentioned before, the moment of actually having to face and realize the decision to seek death was still far away for most of the petition’s signatories. Going against the views of family and others in signing the petition thus had no direct practical or dramatic consequences and was therefore a position that was easier to maintain. Van Wijngaarden and colleagues’ respondents, on the other hand, who were near the possible end of their lives, were more inclined to take the objections and struggles of their children seriously. Furthermore, for their children it was much more complicated, as it was no longer about a parent’s non-binding opinion but about a real and acute death wish.

### The Views and Claims of Physicians

The third category of actors involved in decision-making about ‘completed life euthanasia’ are physicians, as in the Netherlands they are the ones who have to perform euthanasia. A qualitative research project among 28 SCEN doctors^2^ demonstrates that most of the doctors found it undesirable that the existing euthanasia law would be amended to include requests based on ‘completed life’ (Schothorst, 2015). Several doctors feared a slippery slope: growing demands to doctors by people asking for completed life euthanasia, while doctors cannot or do not want to help them. In their view, the general pressure on doctors from patients and their relatives to grant euthanasia has increased in recent years, and a legitimization of the demands on the basis of ‘completed life’ was not considered desirable (Schothorst, 2015).

It seems that a majority of Dutch physicians is highly reluctant when it comes to providing medical aid in dying in cases of ‘completed life’ for several reasons. First, since being tired of living predominately involves psychosocial or existential suffering (and is related to, for example, perceived dependency, fear of future decline and loss of dignity) rather than physical suffering, physicians are less likely to label the suffering as unbearable (; Bolt et al., 2015; Van Tol et al., 2010). Next, they doubt the desperateness of the suffering and wonder whether there are reasonable alternatives to reduce it (Van Delden et al., 2011). Furthermore, many physicians view age-related complaints, the decline of bodily and cognitive function and loss of control as a ‘normal part of life’ (Van Delden et al., 2011), and they tend to perceive tiredness of life and loneliness as mainly social rather than medical problems (Schnabel et al., 2016, p. 122).

New legislation that would regulate the assessment and execution of euthanasia requests outside of current practice was, however, considered interesting by some doctors. In their view, it could perhaps offer the patient a way out ‘without an active role for physicians’ (Schothorst, 2015, p. 20). The advisory committee mentioned above, however, not only rejected the idea of amending or extending the existing euthanasia law, but also did not take up the suggestion that another law could perhaps make euthanasia without the involvement of medical doctors possible. Indeed, they stated that for the sake of safety and due care, it is important that a doctor is involved in performing euthanasia. Only a physician is regarded as capable of judging whether the euthanasia request is voluntary and well-considered. Assessing possible alternatives or treatments is also seen as the task of a doctor (Schnabel et al., 2016, p. 15).

## Discussion

We have sketched out, compared and contrasted the various and often conflicting views and experiences of three groups of actors regarding the right response to older people’s wish to die based on the idea of having lived a ‘completed life’. These three ‘parties’ are the older people themselves, their relatives and friends and the physicians who are the gatekeepers and executers of euthanasia. Obviously, in all three groups, the actors form their views on the basis of their specific position in the debate about ‘completed life euthanasia’. For the older people with a death wish, their position is based on their personal experience of suffering: tiredness of life, loss of meaning and pleasure in life, loneliness and fear of future decline and total dependency. While they were determined to end their lives at a self-chosen moment, they nevertheless also struggled with a constant sense of being torn (Van der Geest, & Satalkar 2019). Standing at the threshold of taking the final step made them more cautious and ambivalent. The signatories of the ‘Of Free Will’ petition had an anticipated wish to die, and by supporting ‘completed life euthanasia’, they intended to safeguard themselves from a fearsome future. Since they based their views on an imagined future, and were not yet facing the realities and complexities of the decision to end their life in the present, they could afford to make their claim straightforward and unambiguous.

The psychology concept of ‘response shift’ offers a credible explanation of people’s change of mind and its impact on their decision-making when they approach the end of life. The terminology of ‘response shift’ refers to how people (usually patients) respond to questions about their quality of life and helps professionals to take suitable clinical decisions. Shifts occur because of various changes within people’s minds as well as in their health condition, their social environment or because of a particular life event (Rapkin & Schwartz, 2004). The most intriguing process that has been observed in this response shift is that patients’ appraisal of their life quality may go up while their physical (or another) condition goes down. Their loss of physical, mental or social ‘capital’ may make what remains more precious. This can be compared to the concept of the ‘disability paradox’ (Albrecht & Devlieger, 1999), while others speak of the ‘aging paradox’ (Kunzmann et al., 2000; Mroczek & Spiro, 2005).

For the purpose of this essay on completed life euthanasia, the possible shift is not so much a response to a researcher, but rather a personal reflection on one’s own condition. Schwartz, who applied the concept to older patients approaching the end of life, pointed out that palliative care clinicians should be cautious when dealing with patients’ advance directives, since they may have changed their minds after discovering that their suffering is ‘*more bearable than expected’* (Schwartz, 2005).

The older people who participated in the researches by Satalkar and by Van Wijngaarden et al. may have faced comparable shifting perspectives regarding old age, due to, for example, pressure from children or a particular event in their lives. Their assumed autonomy to take a decision may dissolve as a result, as is alluded to in the next paragraph.

The second group, relatives and friends, often wanted to respect the will of the older person with a wish to die, and perhaps even did so on a theoretical level. Simultaneously, however, they found themselves in a situation in which they did not want to lose their loved ones yet and consequently considered their wish for euthanasia premature and inconsiderate.

The third group, physicians, currently have the final say in judging the motives of people who request euthanasia. Although doctors recognize and respect the personal reasons of older people in their wish for voluntary death due to ‘completed life’, they might also perceive that the underlying motives are perhaps not well-considered or that the situation does not meet the criteria for due care to justify euthanasia within the current law. For example, they might insist that alternative solutions could still be considered.

This exploratory description of the divergent views and positions of these three groups confuses the older people’s death wishes as well as public debates and policy decisions. More interrelated research is needed to disentangle this clash and better understand the tensions between – and within – these divergent positions.

### Limitations

Although rooted in extensive and profound personal interviews and conversations, this article is limited in its number of respondents and therefore should be seen as exploratory. A larger study that would also involve older people and their relatives as co-researchers and co-authors could provide more robust insights in experiences of older people who consider their lives to be ‘completed’ and wish for a self-directed death. The large recent research, at the request of the Dutch government (Van Wijngaarden et al., 2020), partly fulfils that need, but does not include the views and experiences of children, other relatives and friends of older people asking for completed life euthanasia.

## Conclusion

Our aim was to sketch the complexity of the dilemma with regard to people’s death wish after they feel their life is ‘completed’. As a result of the contradictory views and interests analysed in this article, policy-makers and political authorities find themselves in a muddled situation. They are responsible for the safety and social rules of society as a whole and have to consider all of these divergent perspectives. The resulting decisions will be bound to satisfy some and disappoint others.

As mapped out in the ‘Background’ section above, the advisory committee suggested that the existing law offered sufficient scope to include the majority of requests based on the notion of ‘completed life’, and that more attention should be given to the possibilities of preventing people from becoming tired of life (Schnabel et al., 2016: 15). Preventing tiredness of life, suffering and loneliness in old age is often mentioned in critical reactions to the practice of euthanasia for people who are tired of living (Cohen-Almagor, 2018; Raus et al., 2016). As Van Wijngaarden et al. (2018, p. 419) write:

… the debate on ‘completed life in old age’ should primarily focus not on the question of whether or not to legitimize a self-directed death but on how to build an inclusive society where people may feel less unneeded, useless and marginalized.

The most recent research (Van Wijngaarden et al., 2020) largely agrees with the above quoted conclusion and the recommendations made by the Advisory Committee in 2016 (Schnabel et al., 2016). These conclusions reflect the complexity of the experiences of older people with a death wish, as well as ethical and social concerns in society at large. But they might simultaneously run counter to what the signatories of the ‘Of Free Will’ petition considered their most crucial point: that suffering without being ill should be a valid reason for assisted dying.

Clearly, there just are no easy solutions for death wishes based on the experience of ‘completed life’. To combat the deep existential gravity associated with tiredness of life with social interventions celebrating emancipatory ideals and offering strategies for social participation is not only too easy, it might even be a neglect of the real needs and concerns at stake. As a society, we need to acknowledge that some older persons – however small their number – can indeed find themselves in a world they no longer (want to) understand, without the capacities – or the will – to re-engage with life, not able to throw off the feeling of inertia. Concrete actions, decisions and policies that follow from such an acknowledgement can, however, have several possible forms. Therefore, both political, medical and public commitment is needed to organize a wide and thorough discussion about the death wishes concerned and possible appropriate policies, involving all stakeholders.

To conclude, our reflections are primarily based on developments in Dutch society, but we presume that our reflections can contribute more broadly to related policy discussions on ageing and dying in other Western societies (Raus et al., 2016; Ross, 2015; Span, 2018). Given the emerging facts of longevity and the new demography of death (Leeson, 2014), it is an imperative to nurture new ways of thinking about how we deal with an ageing population in such a way that also the gravity of old age, including experiences of tiredness of life, are recognized. This requires a fundamental cultural debate about the place of old age and death in our society.

## Notes

1. The identities of all participants have been concealed for privacy reasons.

2. The term ‘SCEN doctors’ refers to specially trained physicians who provide physician colleagues with ‘Support and Consultation regarding Euthanasia’. As prescribed in the law, SCEN doctors should be consulted before euthanasia is performed so that they can assess whether they agree that the intended euthanasia case meets the legal criteria.
